# Insights into the radiotherapy-induced deferentially expressed RNAs in colorectal cancer management

**DOI:** 10.22038/IJBMS.2023.71259.15482

**Published:** 2023

**Authors:** Arezu Karimpur Zahmatkesh, Amin Moqadami, Mohammad Khalaj- Kondori

**Affiliations:** 1 Department of Animal Biology, Faculty of Natural Sciences, University of Tabriz, Tabriz, Iran; # These authors contributed eqully to this work

**Keywords:** Colorectal cancer, Non-coding RNAs, Radioresistance, Radiosensitivity, Radiotherapy

## Abstract

Radiotherapy (RT) has been commonly applied to treat advanced local cancers. In radiation therapy, high doses of radiation are utilized to trigger cell death. Radiation often leads to DNA double-strand breakages (DSB), which causes the activation of downstream genes including those for non-coding RNAs (ncRNA) such as long non-coding and RNAsmicro RNAs. The consequence of RT significantly relies on the radiosensitivity of cancer cells, which is affected by multiple factors, including some proteins and cellular processes. Activation of these genes can cause cell cytotoxicity and indirectly damages the cells. Recent studies have shown that non-coding RNAs can play as radiosensitivity or radioinhibitory regulators in cancers by mechanisms such as cell cycle arrest or affecting the DNA damage repair systems. ncRNAs are also known to function as tumor suppressor genes or oncogenes in colorectal cancer and therefore are considered potential diagnostic biomarkers in disease detection. For example, the investigations have shown that miR-29a and miR-224 can be informative biomarkers for early detection or screening of CRC via a noninvasive method such as liquid biopsy.

Here, we discuss ncRNAs involved in the radioresistance and radiosensitivity of CRC and highlight their predictive clinical value in response to RT. Accordingly, this review represents a principal guide in the context of three major types of ncRNAs with potential roles in the pathway of radiosensitivity and radioresistance, including miRNAs, lncRNAs, and circRNAs which can be considered a precious archivement in organizing additional studies and broadening views in this area. Our findings can also assist radiotherapists in predicting CRC patients’ response and, therefore, prognosis to radiation therapy, although, to achieve our goals in the clinic, we certainly need further studies.

## Introduction

CRC (colorectal cancer) is known to be the third and second cause of malignancy and cancer-related mortality worldwide, respectively (1, 2). Based on the Global Cancer Statistics 2020 report, CRC imposes a significant burden on the healthcare system (3). Most cases are diagnosed in the advanced stages of the disease, which implies a need for more effective early diagnosis and treatment strategies (4). Ionizing radiation therapy or radiotherapy is used as a conventional treatment in cancer, either single or combined with other therapeutic approaches. It is an important approach in the management of patients with local advanced gastrointestinal carcinoma such as CRC (5). Tumors differ in sensitivity to radiotherapy and some of them show a high level of radioresistance (6) that impedes their treatment (7). Therefore, identification of factors causing radioresistance or radiosensitivity is critical for diagnosis of responsive patients to improve radiotherapy outcomes.

**Figure 1:**
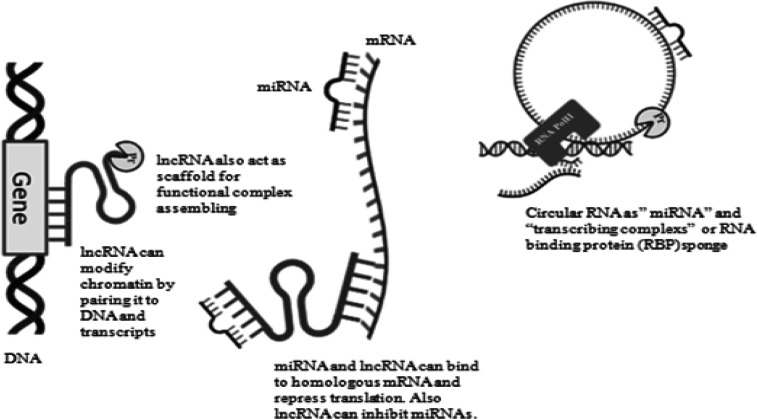
Schematic diagram indicates the functional mechanisms of ncRNAs

 The ENCODE (ENCyclopedia of DNA Elements) project on “junk DNA” has revealed that 90% of the human genome is transcribed to ncRNA, and only 2% is expressed to proteins via mRNA (messenger RNA) translation (8). In this context, recent findings have drawn more attention to studying the ncRNAs and their function (9). According to previous studies, the deregulation of these critical molecules has been detected and determined in the development of different chronic diseases, including coronary artery disease, diabetes, and cancers (10). These types of RNA act by binding to DNA, mRNA, proteins, and also other ncRNAs. Figure 1 represents the functional mechanisms of ncRNAs. MicroRNA (miRNA)s are known as short non-coding RNAs with a minimum of 21 and maximum of 23 nucleotides that often act via targeting mRNAs and also other ncRNAs such as lncRNAs and circRNAs and can cause translational suppression and, in this way, affect different biological processes. LncRNAs include transcripts with 200 nucleotides or more, similar to mRNAs, but they do not code proteins (11) and function by regulating biological processes via interacting with proteins and RNAs (12). circRNAs, another type of ncRNAs, are known as miRNAs sponges, mediators of alternative splicing, and regulators of parental gene expression (13).

Generally, ncRNAs reveal their effects by affecting numerous biological processes, including chromatin remodeling, gene silencing, mRNA processing, and regulation of transcriptional or translational paths.

It has recently been examined and reported that the expression profile of ncRNAs differs between radioresistance and radiosensitive cancerous mass in response to radiotherapy (14). Besides, some ncRNAs can alter the radiosensitivity of the treated cells through different mechanisms (Table 1) and provide valuable information about the prognosis of radiosensitivity or radioresistance (10). Identification of these ncRNAs is very critical in the radiotherapy of cancers.

Here, we focused on the non-coding RNAs which are induced following radiotherapy of CRC rendering radioresistance or radiosensitive, and highlighted their potential as biomarkers for responsiveness to radiotherapy.

***Methods***

The Pubmed databases and Google Scholar were explored to discover research addressing the target ncRNA in CRC radiosensitivity/radioresistance, both *in vitro* and *in vivo* trials, from January 2003 to February 2023. We established and implemented this investigation without considering geographical, racial, or linguistic differences and searched articles using terms including “colorectal cancer” AND “non-coding RNA” AND “radioresistance” AND “radiosensitivity”. 

***Functional mechanisms of radiation therapy***

Radiotherapy is known to either cause forward DNA damage via ionizing radiation or indirectly induce ROS formation, and in this way, harm DNA molecules. Various forms of DNA damage, such as DSBs (double‐strand breaks), SSBs (single‐strand breaks), and AP (abasic sites), can cause cancer cell death, but DSBs are the most destructive.

***Radiation therapy acts efficiently via DNA double-strand breakages (DSBs)***

**Figure 2:**
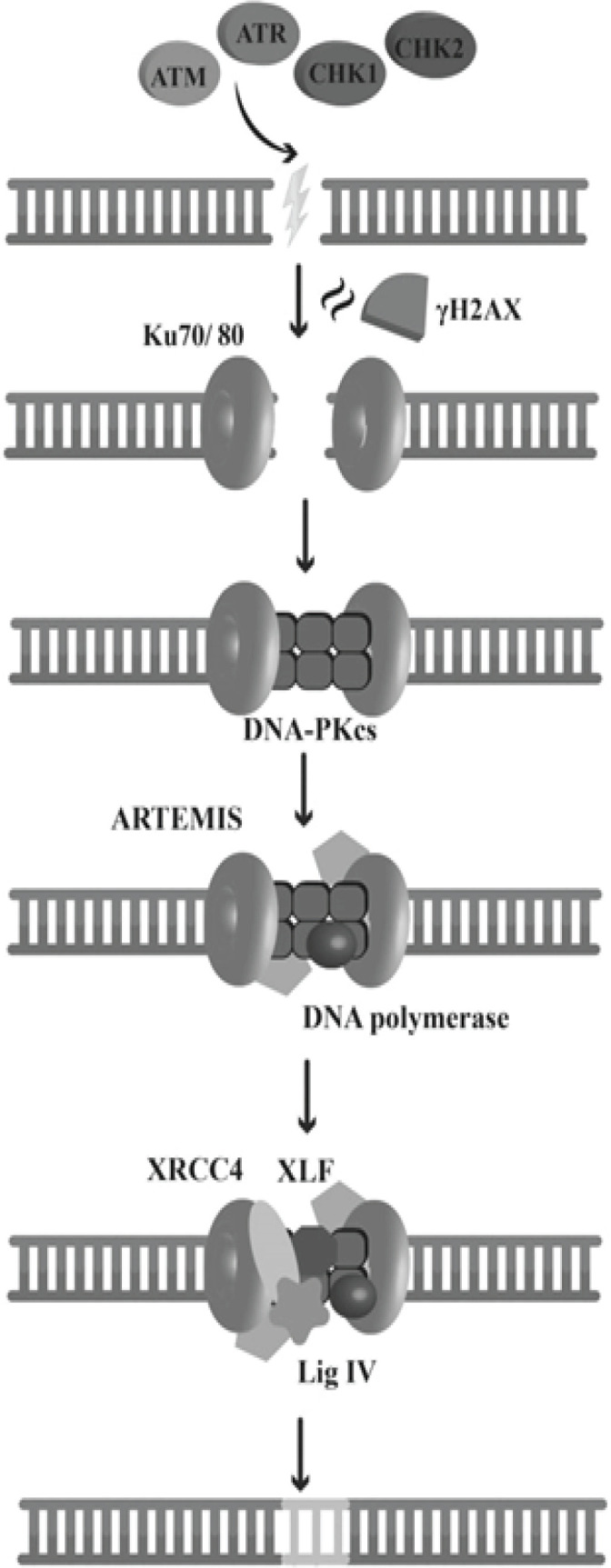
NHEJ (Non-homologous end joining) is the usual mechanism of DSBs repair in cancerous cells, a most destructive DNA injury following ionizing radiation

Following ionizing radiation, DSBs are formed in the DNA of cancerous cells. These breakages are not often repaired and can cause chromosomal aberrations, genomic instability, and finally cell death (15). Cells have two main mechanisms fot repairing double-strand breakages: NHEJ (the non-homologous end joining) and HR (homologous recombination) paths (16). In NHEJ, the ends of broken DNA are joined together directly, while in HR, homologous DNA templates from the sister chromatids are known and ligate together via recombination (17). HR repairs DSBs precisely but the NHEJ pathway is known as an error-prone procedure. Cancerous cells use NHEJ to repair DSBs that arise during an ionizing radiation (18) (Figure 2). In this pathway, several proteins play key roles in distinguishing (CHK1, CHK2, ATM, ATR), signaling (γH2AX complex), and repairing of breaks (DNA-PKcs, Ku80, BRCA1, BRCA2) (15, 16). NHEJ is initiated with Ku70–Ku80 heterodimer binding to the broken DNA molecule ends (19). Subsequently, the catalytic subunits of DNA-PKcs (DNA-dependent protein kinase) are activated, and phosphorylate nuclease enzymes such as Artemis (20, 21) DNA polymerase replace the affected bases, and finally, blunt ends of DNA are ligated by the XLF-XRCC4-DNA ligase IV complex (22). NHEJ is an error-prone mechanism that introduces some errors in the repaired DNA sequence. Accumulation of such errors throughout the genome is fatal for the treated cells. Not only RT affects cancerous cells by introducing DSBs but also it injures them via generating the reactive oxygen species (ROS), indirectly (23). 

***Application of non-coding RNAs in biodosimetry***

Biodosimetry uses physiological, chemical, or biological markers to measure the effects of ionizing radiation on human tissues. These markers have been established by examining biological changes such as chromosomal, molecular, proteomic, or other physiological changes in response to ionizing radiation (24). Studies in the field of the discovery of sophisticated biodosimetry markers make use of genomics, proteomics, metabolomics, cytogenetics, electron paramagnetic resonance, lymphocyte kinetics, and transcriptomics to identify new markers (Table 1). ncRNAs, such as RNA biodosimetry markers, present some benefits over the traditional techniques, for example, dicentric chromosome assay (DCA) (25). DCA depends on culturing lymphocytes *ex vivo* and so is time-consuming which is considered a disadvantage due to decreased efficacy (26). In comparison, ncRNAs could significantly shorten the time of work because of assaying by RT-PCR. Besides, these RNAs are abundant in body fluids such as the bloodstream and are more stable in the fluids, so they might be considered potential biomarkers in different conditions (20, 21). Some of the ncRNAs, including lncRNAs, have been applied in dosimetry, such as *GAS5, TUG1*, and *HOTAIR* which have several targets represented in some literature. In a recent study, irradiation with more than 8 Gy was associated with up-regulation of the* HOTAIR* gene in breast cancer cell lines as a result of radioresistance through affecting miR-218 and miR-449b-5p. In this research knockdown of HOTAIR has been associated with increased cell sensitivity to irradiation via sponging of miR-218. 

Furthermore, another study has indicated sponging the miR-449b-5p via *HOTAIR* enhances radioresistance. In this path inhibition of miR-449b-5p facilitates the expression of downstream chaperone protein HSPA1A (22). Other studies in this field are shown in the Table 2.

***Function of ncRNAs in radiotherapy of CRC***

Due to the critical roles of ncRNAs in disease development and progression, researchers started to study the function of ncRNAs in radiation response. Previous studies have shown that several chemotherapeutic agents can affect the behavior of healthy and cancerous cells in response to radiotherapy (37). These observations have led researchers to consider the same role for ncRNAs. Mainly, several investigations were carried out to recognize how noncoding RNAs can improve radiation response in cancerous cells and protect normal cells from ionizing radiation harm (38). It is now apparent that cells’ response, differentially to ionizing radiation, presents opportunities for radiotherapy combined with other therapeutic agents (39). Based on these studies, the RT-induced differences in miRNA expression patterns rely on dose and fractionation. Researchers have pointed out that non-coding RNAs can be considered key regulators of both radiosensitivity and radioresistance in cancers. 

In addition radiation can influence tumorous cells directly, it can also change the tumor microenvironment (TME) and affect anti-tumor immune responses through increased transcription or protein activation of some genes in targeted cells (40). Currently, microRNAs are known to be a radiosensitivity-predicting factor in colorectal cancerous cells. Some microRNAs in the tumor microenvironment bind to the target genes involved in apoptosis or damage repair path and promote the RT efficiency (i.e., DSBs and apoptosis), therefore sensitizing CRC cells to the RT (41). Furthermore, some studies ascribed improving the radiosensitivity to G2/M phase arrest via specific miRNAs (42). Besides, some lncRNAs enhance the radiosensitivity of CRC cells after irradiation via GSDME-mediated pyroptosis (as a type of programmed necrosis). It has been indicated that *GSDME* works as a potent tumor suppressor gene in a large fraction of gastrointestinal malignancies like gastric and colorectal tumors (43). 

Collectively, mechanisms of radiosensitivity following RT in CRC cells can be categorized into several ways such as promoting cell apoptosis, DNA damage, and cell pyroptosis while inhibiting cell cycle progression, cell stemness, autophagy, and Epithelial-Mesenchymal Transition (44) (Table 3). On the contrary, non-coding RNAs in the tumor microenvironment can make cancerous cells resistant to RT too (44). They promote radioresistance by inhibiting tumor suppressor gene signaling pathways or activating oncogenic signaling (45). Some non-coding RNAs act by suppressing apoptosis or promoting cell proliferation and DNA damage repair (46) or promoting cell growth and autophagy. In total, the mechanisms of ncRNA-mediated radioresistance of colorectal cancer can be summarized as inhibition of cell apoptosis and DNA damage, while promoting cell autophagy, EMT, and cell cycle transition (44) (Table 3).

***Non-coding RNAs enhance radiosensitivity***

As direct or indirect targets in RT and colorectal cancer, miRNAs have been extensively studied. By inducing colorectal cancer cells to acquire irradiation-induced apoptosis, MiR-185 was shown to increase radiosensitivity (47). Ji *et al*. claimed that an increase in miR-15b improved the susceptibility of CRC cells to RT by preventing metastasis and cell proliferation and that miR-15b was substantially down-regulated in CRC tissues (48). Through a number of biological pathways, researchers showed that overexpression of  miR-140-5p and miR-506-3p greatly increased the radiosensitivity of CRC cells (49). MiR-124 was found at reduced concentrations in CRC cell lines and tissues, but larger concentrations of this miRNA made CRC cells more sensitive to RT (50). The sensitivity of CRC cells to RT was increased through miR-519b-3p’s stimulation of irradiation-induced apoptosis (51). It has been observed that the tissues of patients with CRC who reacted to RT exhibited elevated levels of miR-519b-3p, miR-21-5p, and miR-214. Additionally, miR-214 was found to inhibit irradiation-induced autophagy both *in vivo *and* in vitro*, while also enhancing the sensitivity of CRC cells to RT (52). By preventing K-Ras activity, inhibition of let-7a decreased the responsiveness to RT in CRC cells expressing wild-type TP53 (53). CRC patients’ tissues with partial RT responses showed raised levels of miR-451a, and by inhibiting cell development and affecting the survival of cells, overexpression of mentioned miRNA increased the radiosensitivity of CRC cells (54). In radioresistant cell lines, down-regulation of let-7g, miR-320a, and miR-132 utilizing microarray analysis and qRT-PCR is reported, however, overexpression of let-7g, miR-320a, and miR-132 led to significant increase of the radiosensitivity of CRC cells (55). Furthermore, it has been shown that Let-7e increases the radiosensitivity of CRC cells by enhancing irradiation-induced apoptosis and inhibiting cell growth and cell cycle transition (38). In contrast to miR-100’s down-regulation in CRC cell lines and tissues (56), through promotion of radiation-induced apoptosis and inhibition of DNA damage repair, miR-100 overexpression greatly increased the radiosensitivity of CRC cells (57). In the radiation-resistant CRC cell lines, miR-630 expression was reduced. The RT-induced cytotoxicity of CRC cells was enhanced by up-regulated miR-630, which also enhanced their sensitivity (58). SNAI1-mediated stemness is inhibited by miR-145, which increases the radiosensitivity of CRC cells (59). 

**Figure 3:**
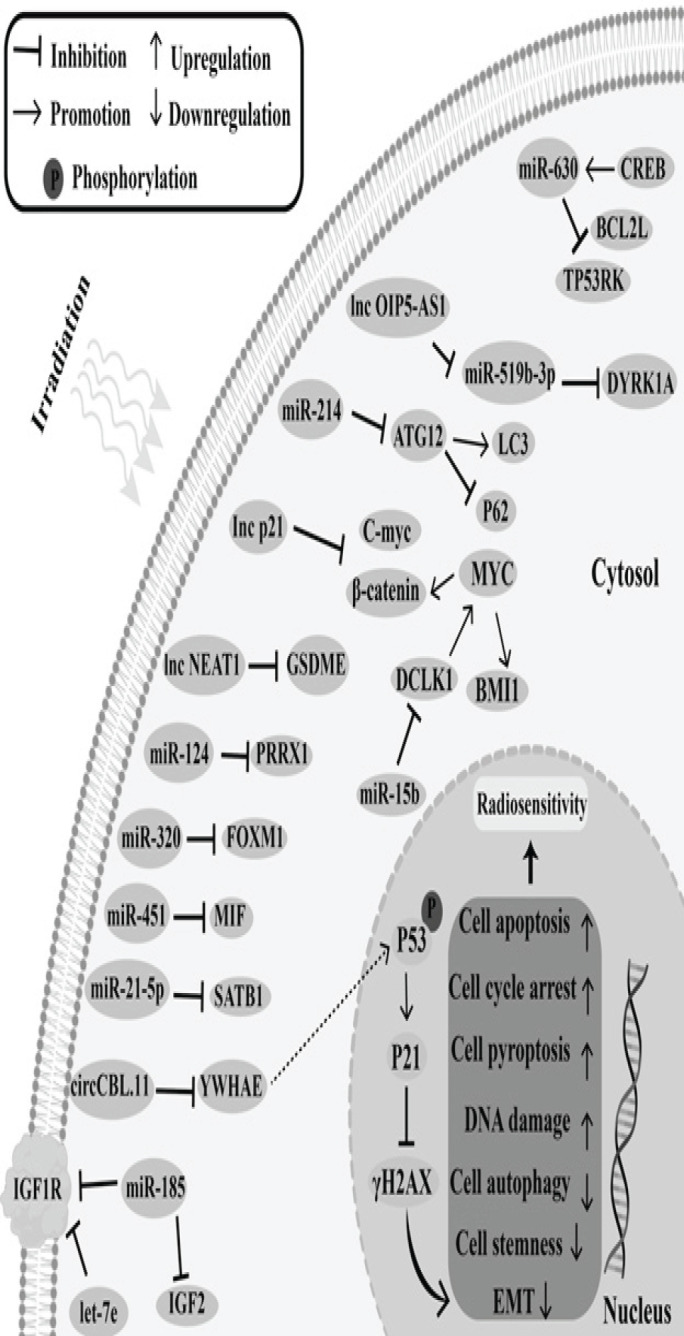
Schematic diagram representing some known ncRNAs and mechanisms by which they could enhance radiosensitivity of colorectal carcinoma after radiotherapy

Additionally, critical roles for circRNAs and lncRNAs in colorectal cancer and RT are increasingly being identified. In both CRC cell lines and tissues, lower and higher levels of lnc-p21 increased the susceptibility of CRC cells to RT, respectively (60). Inhibiting cell growth *in vitro*, circ-CBL.11 up-regulation boosted the responsiveness of colorectal cancer cells to RT (61). By intensifying the pyroptosis caused by radiation exposure, lnc-NEAT1 made CRC cells more sensitive to RT (62). Microarray analysis and qRT-PCR were utilized to show that lnc-OIP5-AS1 was suppressed in colorectal cancer cells resistant to radiotherapy, despite a finding that raising the level of lnc-OIP5-AS1 substantially raised the sensitivity of colorectal cancer cells to radiotherapy (63). Figure 3 and Table 4 summarize several ways in which non-coding RNAs may improve the radiosensitivity of CRC.

***Non-coding RNAs induce radioresistance***

**Figure 4:**
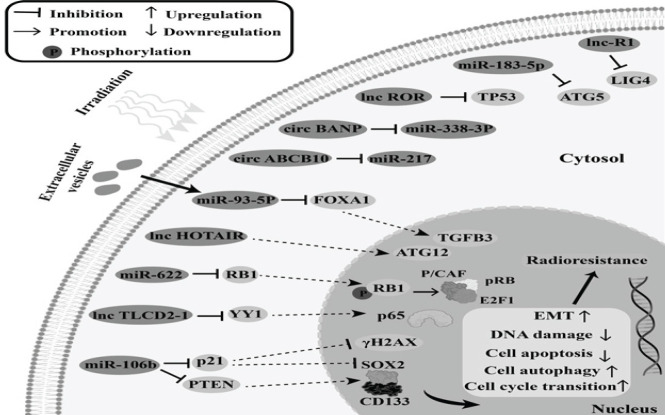
Schematic diagram representing some known ncRNAs and mechanisms by which they could induce radioresistance in colorectal carcinoma after radiotherapy

The association between ncRNAs and radioresistance after radiotherapy of carcinoma has been found to be substantial in several studies. MiR-93-5p was shown to be elevated in CRC tissues and improve RT resistance in CRC cells by promoting cell proliferation and preventing irradiation-induced apoptosis (68). Inducing radioresistance in CRC cells, miR-222 and miR-155 promote cellular proliferation and DNA damage repair (69). Furthermore, researchers discovered that lnc-RI increased cell viability, repaired DNA damage, and prevented irradiation-induced apoptosis, all of which substantially decreased the susceptibility of CRC cells to RT (70). After RT, lnc-HOTAIR levels in patient serum, cell lines, and CRC tissues were all noticeably increased. In addition, *in vitro *and* in vivo*, lnc-HOTAIR promoted cell proliferation and autophagy while inhibiting irradiation-induced apoptosis, resulting in radioresistance (71). The levels of miR-155, miR-222, and lnc-00152 were elevated in radioresistant cell lines of CRC. The migration and invasiveness of CRC cells were markedly suppressed by lower levels of lnc-00152 in radioresistant cells (72). Following miR-622 overexpression, CRC cells exhibited *in vitro* resistance to RT (71). CRC cells were less sensitive to RT *in vitro* when miR-224 was overexpressed (55). Moreover, CRC and intestinal cells showed irradiation resistance as a result of elevated miR-29a levels (73). In CRC cell lines and tissues, there were significantly increased levels of miR-183-5p, lnc-ROR, circ-ABCB10, and circ-BANP (74). By increasing cell proliferation and stimulating EMT, circ-ABCB10 made CRC cells more radioresistant (75). Moreover, by restricting cell proliferation and boosting irradiation-induced apoptosis, the reduction of lnc-ROR decreased the tolerance of CRC cells to RT (76). MiR-183-5p triggered cell proliferation and increased cell survival both *in vivo* and *in vitro*, which reduced CRC cells’ resistance to RT (77). By increasing the cell survival percentage and promoting cell autophagy, circ-BANP decreased the sensitivity of CRC cell lines to radiation (78). In CRC tissues of patients treated with RT, there was an increase in Lnc-UCA1. Lnc-UCA1 increased EMT and G2/M arrest while decreasing irradiation-induced apoptosis, which interfered with the radiosensitivity of CRC cells (79, 80). In CRC radiosensitive cell lines and tissues, lnc-TLCD2-1 was down-regulated; according to Yu *et al*. by increasing cell survival and suppressing irradiation-induced apoptosis, lnc-TLCD2-1 caused CRC cells to become radioresistant (64). Highly differentiated CRC cell lines and CRC tissues both showed elevated miR-106b levels. The ability to initiate tumors, the cell survival percentage and DNA damage repair were all increased when miR-106b was overexpressed, which provided radioresistance to CRC cells (81). Therefore, in the sensitivity of colorectal cancers to radiation, ncRNAs play important roles. Some mechanisms by which non-coding RNAs could induce radioresistance in CRC after radiotherapy are summarized in Figure 4 and Table 5. 

***Non-coding RNAs may predict response to the radiotherapy***

The potential of ncRNAs as cancer diagnostic biomarkers or predictors of treatment effectiveness has received considerable attention (90). The prognosis of CRC patients would certainly be improved and personalized treatment would be possible if the response to RT could be accurately predicted by ncRNA profiles. According to Ji *et al*., High levels of miR-15b predicted a positive response to neoadjuvant radiation, while miR-15b was dramatically down-regulated in CRC tissues in comparison to adjacent normal tissue (48). While the expression of lnc-p21 was down-regulated in responder serum, it was up-regulated in CRC tissues. High tissue levels of lnc-p21 expression were found in CRC patients, and these patients responded well to postoperative RT (37). In the radiosensitive CRC patients’ plasma, miR-140-5p and miR-506-3p were elevated, and these patients responded well to RT. Besides, patients with high serum levels of these two miRNAs also showed a strong response to RT. The ability of miR-140-5p and miR-506-3p to distinguish between radiosensitive and radioresistant individuals had a prediction accuracy of 0.925 (49). In contrast to the overexpression of miR-214 in radiosensitive CRC tissues, miR-214 expression in plasma was reduced in CRC patients following RT. Accordingly, increased tissue levels of miR-214 suggested that CRC patients would respond better to radiotherapy (52). Using microarray, a study discovered that in the radioresistant CRC cell lines, fourteen microRNAs were elevated and twenty-two microRNAs were reduced (55). In CRC cells resistant to radiotherapy, Xiong *et al*. found that two circRNAs and five lncRNAs were reduced, while three circRNAs and one lncRNA were elevated (55, 91). Additionally, lnc-NR015441, lnc-NR033374, and lnc-R05532 were shown to have a favorable correlation with the CRC cell lines’ resistance to irradiation (92). As indicated in Table 6, some investigated non-coding RNAs may be regarded as predictive responses to RT.

***Clinical Perspective of Non-coding RNAs***

*Patient’s Survival prediction*

It has been demonstrated that the expression of MiR-15b has a negative association with liver metastases and adverse clinicopathological characteristics in CRC patients. Additionally, neoadjuvant treatment results, disease-free, and overall survival were all significantly reduced in people with low levels of miR-15b (93).

According to follow-up data examined by Liu *et al*., the expression of lnc-HOTAIR was discovered to be inversely linked with the survival of CRC patients (83). The GSE17536 dataset revealed that higher lnc-TLCD2-1 expression in CRC patients was associated with more prevalent overall and disease-specific survival (80). Patients with CRC who exhibited high miR-183-5p expression had lower OS (94). According to research, lnc-p21 served a variety of predictive purposes in CRC patients. Poor OS and DFS have been associated with elevated lnc-p21 levels in CRC patients. Increased lnc-p21 levels were similarly associated with shorter OS in the plasma of CRC patients (95). Low DBET in patients treated with LINC00909 frequently indicated better OS. But in CRC patients, high LINC00909, FLJ33534, and DBET levels frequently identified a poor DFS (96).

***Non-coding RNAs as diagnostic and predictive biomarkers***

LncRNAs are considered applicable biomarkers for diagnosis and prognosis of CRC and prediction of the response to therapy. LncRNAs have several advantages as diagnostic biomarkers in clinical practice, such as ease of detection in body fluids and blood, cell type-specific expression patterns, and fluctuations in expression levels in CRC samples (97). Some examples of lncRNAs that have been reported as biomarkers for CRC are HOTAIR, MALAT1, H19, CCAT1, and XIST. These lncRNAs can be detected by various methods such as RT-PCR, FISH, or NGS 2. LncRNAs can also be used as prognostic biomarkers to stratify patients according to their risk of recurrence, metastasis, or survival. For instance, LncRNAs can also be used as predictive biomarkers to guide the selection of optimal treatment strategies for CRC patients. For example, lncRNA UCA1 can predict the sensitivity of CRC cells to 5-fluorouracil (5-FU), a common chemotherapeutic agent (98). 

***Non-coding RNAs as therapeutic targets***

LncRNAs can be modulated for therapeutic purposes by either inhibiting or enhancing their expression or function. This type of ncRNAs can be inhibited by various approaches such as RNA interference (RNAi), antisense oligonucleotides (ASOs), small molecule inhibitors, or CRISPR-Cas9 system (60). For example, RNAi-mediated knockdown of HOTAIR can suppress CRC cell proliferation, invasion, and migration (71). LncRNAs can also be enhanced by using gene therapy or synthetic mimics. For example, overexpression of lncRNA MEG3 can induce apoptosis and inhibit angiogenesis in CRC cells (99). Moreover, lncRNAs can be used for combination therapy with other agents to improve therapeutic efficacy and overcome drug resistance. For instance, the co-delivery of lncRNA GAS5 and doxorubicin can enhance the antitumor effect and reduce the side effects in the CRC mice model (100). It has been demonstrated that the expression of MiR-15b has a negative association with liver metastases and adverse clinicopathological characteristics in CRC patients. Low DBET usually suggest improved OS in LINC00909-treated patients. High levels of LINC00909, FLJ33534, and DBET, however, generally indicate a poor DFS in CRC patients (96).

Non-coding RNAs often work via cellular process regulation by binding to the proteins, mRNA, DNA, and even other ncRNAs. miRNA usually manifests its effects by attaching to homologous mRNA following the suppression of the translation. LncRNAs function by targeting mRNA or inhibiting miRNA, and also can do their role as a trap of the functional complex. CircRNAs sponge the target miRNA or transcription complex and show their effects

**Table 1:** Fields used in the discovery of biodosimetry markers to confirm the potential genes and proteins which provide the opportunity for use in quick and early evaluation of a significant radiological incident

Genomics
Proteomics
Metabolomics
Cytogenetics
Electron paramagnetic resonance
Lymphocyte kinetics
Transcriptomics

At least three protein complexes function in this way, including a) distinguishing (ATR, Chk1, Chk2 and ATM), b) signaling (γH2AX), and c) repairing (Ku80, DNA-PKcs, BRCA1and BRCA2)

**Table 2:** Some classes of ncRNA including lncRNAs, micro RNAs, and circRNAs have been studied in the field of biodosimetry

**Non-coding RNA**	**Targets of non-coding RNA**	**Source**	**Response to radiotherapy**	**References**
lncRNA TUG1	HMGB1 miR-139-5p	bladder cancer tissue sample/ cell line (SW780, HT1376, BIU87, and T24)	Radioresistance	(27)
lncRNA TUG1	miR-139-5p SMCA1	prostate cancer tissue sample/ cell line (LNCaP, 22RV1, PC-3, DU145) / mouse model	Radioresistance	(23)
lncRNA GAS5	miR-106b miR-205-5p	cervical cancer tissue sample/ cell line (SiHa, ME180)	Radiosensitivity	(24)
lncRNA GAS5	Up-regulation of p21	stomach cancer tissue sample/ cell line (HGC-27, SGC-7901)	Radiosensitivity	(25)
miRNA let-7	RAS	lung cancer cell line (A549)/ *Caenorhabditis elegans* model	Radiosensitivity	(26)
miR-21	PTEN	mouse model of RIPF(Radiation-induced pulmonary fibrosis)	Radioresistance	(28)
miR-221	PTEN	colorectal cancer cell line (HT-29, Lovo, SW-480, and Caco2)	Radioresistance	(29)
miR-521	DNA repair protein CSA	prostate cancer cell line (LNCaP )	Radiosensitivity	(30)
miR-95	sphingolipid phosphatase SGPP1	prostate cancer cell line (PC3)	Radioresistance	(31)
miR-181a	Bcl-2 (an apoptosis regulator)	glioma cell line (U87MG)	Radiosensitivity	(32)
miR-17	MDM2 as negative p53 regulator	glioblastoma cell line (M059J and M059K)	Radiosensitivity	(33)
circRNA_100367	miR-217	esophageal squamous cell line (KYSE-150R and KYSE-150)	Radioresistance	(34)
circPITX1	miR-329-3p	glioma tissue sample/ cell line U251, LN229	Radioresistance	(35)
circTUBD1	miR-146a-5p	hepatic stellate cell line (LX-2 cells)	Radioresistance	(36)

**Table 3:** A) Mechanisms of ncRNA-mediated radiosensitivity in colorectal cancer. B) Mechanisms of ncRNA-mediated radioresistance in colorectal cancer (CRC)

Related to radiosensitivity	Related to radioresistance
**↑cell apoptosis**	↓cell apoptosis
**↑DNA damage**	↓DNA damage
**↓cell autophagy**	↑cell autophagy
**↓Epithelial-Mesenchymal Transition**	↑Epithelial-Mesenchymal Transition
**↑cell pyroptosis and ↓cell cycle transition**	↑cell cycle transition
**↓cell stemness**	

**Table 4:** Some ncRNAs and mechanisms by which they could enhance radiosensitivity of colorectal carcinoma after radiotherapy

**Non-coding** **RNAs**	**Targets of non-coding RNA**	**Expression in CRC**	**Source(s)**	**Mechanism**	**References**
lnc-OIP5-AS1	miR-369- 3p/ DYRK1A	Decreased	Cell line	Impair cell clonogenic survival, stimulate irradiation-induced apoptosis, and enhance radiosensitivity	(63)
lnc-NEAT1	miR-448/ GSDME	Increased	Cell line	Stimulate IR-induced pyroptosis and enhance radiosensitivity	(61)
TLCD-2	miR-193a-5b	decreased	Cell line		(64)
lnc-p21	-	Decreased	Tissue and cell line	Stimulate irradiation-induced apoptosis and enhance radiosensitivity	(49)
miR-451	MIF	Decreased	Tissue	Reduce cell proliferation and sensitize cells to RT	(65)
miR-15b	DCLK1	Decreased	Tissue	Inhibit cell growth, invasion, and metastasis and enhance radiosensitivity	(48)
miR-140-5p and miR-506-3p	-	Increased	Serum	Reduce cell production, survival rate, and enhance radiosensitivity	(49)
miR-124	PRRX1	Decreased	Tissue and cell line	Stimulate irradiation-induced apoptosis, prevent EMT, and cell stemness, and enhance radiosensitivity	(50)
miR-214	ATG12	Decreased	Serum and cell line	Inhibit IR-induced autophagy and enhance radiosensitivity	(52)
Let-7a	-	-	-	Inhibit cell growth and enhance Radiosensitivity	(66)
let-7g, miR-320a and, miR-132	-	-	-	Enhance radiosensitivity	(55)
let-7e	IGF-1R	-	-	Arrest cell cycle transition, promote apoptosis, and enhance radiosensitivity	(48)
miR-100	-	Decreased	Tissue and cell line	Stimulate irradiation-induced apoptosis and DNA double-strand breaks, and enhance radiosensitivity	(67)
miR-630	BCL2L2 and TP53RK	Decreased	Cell line	Enhance irradiation-induced cytotoxicity and enhance radiosensitivity	(58)
miR-145	-	Decreased	Cell line	Inhibit cell stemness and enhance radiosensitivity	(59)
miR-185	IGF1R and IGF2	-	-	Stimulate irradiation-induced apoptosis	(47)
circ-CBL.11	miR-6778- 5p/YWHAE	Increased	Cell line	Suppress cell proliferation	(61)

By inhibiting (┬) and promoting (↑) some gene expressions in radiotherapy-induced cells, ncRNAs could affect their up-regulation and down-regulation; consequently, increasing cell apoptosis, cell cycle arrest, cell pyroptosis, and DNA damage and decreasing cell autophagy, cell stemness, and EMT could lead to radiosensitivity

**Table 5:** Some ncRNAs and mechanisms by which they could induce radioresistance in colorectal carcinoma after radiotherapy

**Non-coding** **RNAs**	**Targets of non-coding RNA**	**Expression in CRC**	**Source(s)**	**Mechanism**	**References**
lnc-RI	miR-4727-5p/ LIG4	Increased	Serum, tissue and cell line	Simplify cell growth and cell cycle transition, repress radiation-induced apoptosis, and induce radiation resistance	(82)
lnc-HOTAIR	MiR-93/ATG12	Increased	tissue and cell line	Facilitate cell viability and cell autophagy, repress radiation-induced cell apoptosis, and induce radiation resistance	(83)
lnc-HOTAIR	-	Increased	Serum, tissue and cell line	Promote cell division, invasion, and migration, inhibit radiation-induced apoptosis, and induce radiation resistance	(71)
Lnc- TTN-AS1	miR-134-5p	Increased	Cell line		(84)
lnc-ROR	p53/miR-145	Increased	tissue and cell line	Promote cell viability, inhibit radiation-induced apoptosis, and induce radiation resistance	(85)
lnc-UCA1	-	Increased	tissue and cell line	Promote cell proliferation, cell cycle transition and EMT, inhibit radiation-induced apoptosis, and induce radiation resistance	(80)
lnc-TLCD2-1	miR-193a- 5p/YY1	Increased	tissue and cell line	Promote cell proliferation, inhibit radiation-induced apoptosis, and induce radiation resistance	(64)
LINC00152	-	Increased	Cell line	Facilitate cell division, invasion, migration, and promote radiation resistance	(86)
miR-93-5p	FOXA1	Increased	Tissue	Facilitate cell proliferation, inhibit radiation-induced apoptosis, and promote radiation resistance	(68)
miR-224	-	Increased	-	Induce radiation resistance	(55)
miR-155 and miR-222	-	Increased	Cell line	Facilitate cell proliferation and induce radiation resistance	(68)
miR-183-5p	ATG5	Increased	tissue and cell line	Enhance cell viability and survival fraction, and induce radiation resistance	(77)
miR-29a	PTEN	Increased	tissue and cell line	Increase surviving fraction and induce radiation resistance	(87)
miR-106b	PTEN and p21	Increased	tissue and cell line	increase the ability of tumor-initiating cells, DNA damage repair and cell survival rate, and induce radiation resistance	(81)
circ-ABCB10	miR-217	Increased	tissue and cell line	Stimulate cell proliferation, migration, invasion, and induce radiation resistance	(75)
circ-BANP	miR-338-3p	Increased	tissue and cell line	Increase cell viability, cell survival fraction and cell autophagy, and induce radiation resistance	(78)
circ_IFT80	miR-296-5p/MSI1	increased	Cell line	stimulated tumor growth *in vivo*, facilitate tumorigenesis, and induce radioresistance	(74)

The effect of irradiation on ncRNAs and altering the expression of some genes, subsequently increasing EMT, cell cycle transition, and cell autophagy, and decreasing DNA damage and cell apoptosis could enhance radioresistance

**Table 6:** Some predictive non-coding RNAs in the field of radiation

**Non-coding** **RNAs**	**Expression**	**Source(s)**	**Predictive assessment**	**References**
lnc-p21	High	Tissue and serum	CRC patient with high expression of lnc-p21 in tissue displays a good response to postoperative CRT	(85)
miR-140-5p and miR-506-3p	High	Serum	Patient has a positive response to RT and has high expression of miR-140-5p and miR-506-3p. To discriminate between individuals who are radiosensitive and radioresistant, the miR-140-5p and miR-506-3p AUC values were 0.925	(49)
miR-214	High	Tissue and serum	A patient who has high tissue levels of miR-214 has a positive response to RT	(52)

## Conclusion

Radiotherapy is one of the main conventional therapeutic methods in advanced CRC cases, but its usefulness is restricted due to radioresistance. The precise mechanisms of radioresistance and radiosensitivity have not been completely elucidated, and overcoming this challenge is considered a priority in cancer research. According to the findings in the role of ncRNAs in tumorgenesis and responsiveness to various therapeutic methods, their classification into tumor suppressor genes and oncogenes is logical. Based on studies in this area, non-coding RNAs, especially mentioned RNAs, contribute as components of a complicated regulatory network responding to radiation injury. This network functions through various mechanisms, including interactions with DNA sequences, mRNAs, non-coding RNAs, and also proteins that can modulate cellular processes such as cell apoptosis, DNA damage repair, cell autophagy, cell pyroptosis, stemness capacity, epithelial-to-mesenchymal transition (EMT), and cell cycle transition, following the radiation. Furthermore, functional analyses of ncRNAs via methods including RNA immunoprecipitation (RIP), FISH, NGS, and others have presented further information about the relation between ncRNA and tumor staging, response to conventional treatment, and prognosis. Besides, some clinical trials are proceeding to determine the potential role of non-coding RNAs, such as long non-coding RNA CCAT1 (ClinicalTrials.gov Identifier: NCT04269746) and also miR-31(ClinicalTrials.gov Identifier: NCT03362684) in CRC individualized medicine. 

In this review, we outlined the lncRNAs, miRNAs, and circRNAs that are known to enhance radiosensitivity or induce radioresistance in CRC. The information presented in this study can serve as an updated archive for designing and implementing further studies in this field. Additionally, we evaluated the probable predictive significance of mentioned ncRNAs in response to radiation therapy, and the results emphasize the advantages of these findings as guidance in the context of CRC individualized treatments in the clinic. However, more examinations are required to study the practical importance of non-coding RNAs in colorectal cancer radiation therapy until we can present a comprehensive database. 

## Authors’ Contributions

A KZ, A M, and M KK designed the study. A KZ and A M collected data and wrote the manuscript. M KK supervised, directed, and managed the study. A KZ, A M, and M KK approved the version to be published. 

## Conflicts of Interest

The authors declare no potential conflicts of interest. 
